# Atlanta Medical College

**Published:** 1871-04

**Authors:** 


### ATLANTA MEDICAL COLLEGE.

This Institution is now about entering upon its thirteenth regular Course of Lectures, and with flattering prospects of a class larger than usual.

This is indeed encouraging, in view of the pecuniary embarrassment which now exists through the South. There is evidently less surplus of money, and less ability on the part of those desiring to prosecute professional studies, than was found twelve months ago. Notwithstanding this, the correspondence with students, up to this time, shows a decided increase of interest manifested for the course of 1871, which opens on the first Monday in May.

This, like all other medical and literary institutions, particularly in the South, suffered from the war to, at least, half the amount of its former patronage. To medical colleges, the state of the country, resulting from this cause, proves peculiarly embarrassing. In addition to the great stringency in money matters, the demand for physicians and the prospect of reward for their services have greatly diminished under the new ordor of things. These difficulties in the way of accessions to the medical profession, however, are rapidly giving way, from a more settled condition of the country. Even now, in many parts of this and other States, there is pressing demand for reliable physicians ; and in communities, too, that are responsible in every respect.

The ensuing course of lectures in the Atlanta Medical College will be found complete in every particular, as will be seen by reference to the advertisement, found in this issue of the Journal.

The Faculty, as now constituted, shows the return of members identified with the College at its organization in 1855, but closed their connection with the suspension of exercises at the commencement of the war in 1861. The Trustees, at their meeting in September last, called to the Chairs of Practice and Anatomy, the original occupants. The names of John W. Jones and H. W. Brown, so prominently connected with the early history of the College are, doubtless, held in plea ant remembrance by many who, during their papilage, were cheered on the rugged course of study by their veterans of medical science.

Altogether the ensuing course promises to be the most successful and pleasant had in the College since the war.

A preliminary course will be given gratuitously, during the month of April, on Chemistry, Clinical Medicine, Surgery, and Anatomy. To this, all students are invited, who desire to devote a few weeks to such studies before the commencement of lectures in May.

